# LIFESPAN AGE-RELATED DIFFERENCES IN THE REGIONAL WHITE MATTER MICROSTRUCTURE OF THE HUMAN CORPUS CALLOSUM

**DOI:** 10.1093/geroni/igz038.3435

**Published:** 2019-11-08

**Authors:** John Lynn, Chaitali Anand, Muzamil Arshad, Dalal Khatib, Jeffrey Stanley, Naftali Raz

**Affiliations:** 1 Wayne State University, Detroit, Michigan, United States; 2 Saint Joseph Hospital, Chicago, Illinois, United States

## Abstract

The corpus callosum (CC) connects homologous cortical structures across hemispheres and is the largest white matter tract in the human brain. Post-mortem studies suggest that CC myelination begins in infancy, reaches a plateau in the middle age, and declines in the later years. The latter is accompanied by myelin disruption and reduction in fiber density and diameter, i.e. changes in intra-/extracellular water space. We used multi-echo T2 imaging to estimate, via multi-exponential T2 relaxation of water, the myelin water fraction (MWF), a direct proxy for myelin content, and geometric mean T2 (geomT2IEW) that reflects water in the intra-/extracellular space, to investigate age differences in five CC regions covering its anterior to posterior span in 395 healthy individuals (7-85 years; 161M+235F). The general linear model analysis of MWF showed main effects of age and age-squared conditioned on interactions by CC region. Univariate polynomial regressions on three age groups (7-29, 30-59, and 60-85 years) revealed the overall quadratic association between age and MWF as mainly driven by the positive linear relationship in the youngest group and minimal differences in the remainder of the lifespan, save for two weak negative linear associations in the anterior/middle CC body. With geomT2, a main linear effect of age, and significant interactions between age and age-squared by region were observed. The positive linear association was especially prominent in the regions with greater fiber density. The results are consistent with CC myelination into adulthood and decreased axonal density and diameter but not prominent myelin degeneration in elderly individuals.

